# Diet Macronutrients Composition in Nonalcoholic Fatty Liver Disease: A Review on the Related Documents

**DOI:** 10.5812/hepatmon.10939

**Published:** 2014-02-17

**Authors:** Ali Hashemi Kani, Seyed Moayed Alavian, Fahimeh Haghighatdoost, Leila Azadbakht

**Affiliations:** 1 Food Security Research Center, Isfahan University of Medical Sciences, Isfahan, IR Iran; 2 Department of Community Nutrition, School of Nutrition and Food Science, Isfahan University of Medical Sciences, Isfahan, IR Iran; 3 Middle East Liver Diseases Center (MELD), Tehran, IR Iran

**Keywords:** Non-alcoholic Fatty Liver Disease, Food, Intra-Abdominal Fat, Carbohydrates, Proteins

## Abstract

**Context:**

Non-alcoholic fatty liver disease (NAFLD) is a growing health problem in both developed and developing countries. Metabolic abnormalities, specially insulin resistance and hyperglycemia are highly correlated with NAFLD. Lifestyle modifications including physical activity and promoting nutrient intakes are critical in prevention and treatment of NAFLD. Hence, in this article we aimed to review the evidence regarding the effects of various macronutrients on fat accumulation in hepatic cells as well as the level of liver enzymes.

**Evidence Acquisitions:**

The relevant English and non-English published papers were searched using online databases of PubMed, ISI Web of Science, SCOPUS, Science Direct and EMBASE from January 2000 to January 2013. We summarized the findings of 40 relevant studies in this review.

**Results:**

Although a hypocaloric diet could prevent the progression of fat accumulation in liver, the diet composition is another aspect which should be considered in diet therapy of patients with NAFLD.

**Conclusions:**

Several studies assessed the effects of dietary composition on fat storage in liver; however, their findings are inconsistent. Most studies focused on the quantity of carbohydrate and dietary fat; whilst there is very limited information regarding the role of protein intake.

## 1. Context

Non-alcoholic fatty liver disease (NAFLD) is identified as lipid accumulation in individuals who consume less than 20g ethanol/d and not affected by other known causes of fatty liver such as drugs consumption or toxins exposure (1). The fat content of a normal liver is less than 5% by its weight; while becomes 50-80% in fatty liver diseases (2). NAFLD includes a range of diseases from simple steatosis, to inflammatory steatohepatitis (NASH, the most severe form of NAFLD) with increasing fibrosis, to cirrhosis and end-stage liver disease (3). In Western countries, NAFLD prevalence is reported 20–30% in the general adult population (4). The prevalence of this disease is different from 2.8% to 24% (5, 6) and it is near 10% in developing nations (7). However, 31% prevalence is reported from different ethnicity all over the USA (5). According to the report of Iranian Ministry of Health, this disease is responsible for the mortality of 1% of people older than 15 years (8). The pathogenesis of NAFLD is complicated. The suggested mechanism underlying the fatty liver is the “two-hit hypothesis”. The first hit is fat accumulation in the liver, which impairs fatty acids metabolism including de novo lipogenesis, β-oxidation, triacylglycerol clearance and very low-density lipoprotein (VLDL) extraction, by its own. The second hit is probably due to pro-oxidative and hepatotoxic events (9). Furthermore, metabolic syndrome, diabetes, obesity and central adiposity are tightly correlated with this disease. Insulin resistance, inflammation and oxidative stress are mainly involved in the disease progression (10). In most patients, insulin resistance is a good predictor of NAFLD and NASH risk (11). Also, elevated serum level of tumor necrosis factor-α (TNF-α) is considered as a manifestation of NAFLD (12). Another probable mechanism for NAFLD is the overgrowth of small intestinal bacteria (13-15).

The primary treatment of NAFLD is life style modifications due to its important role, both in prevention and maintenance of overall health for patients with NAFLD (10). Among life style interventions, dietary intake has been encountered with high emphasis. Total energy intake, macronutrients and different micronutrients especially antioxidants, have been linked to the development or prevention of NAFLD (16). Metabolic effects of Polychlorinated biphenyls (PCBs), associated with abnormal liver enzymes and suspected NAFLD, are highly related to macronutrient interactions with high fat diet. Antioxidant depletion might be an important consequence of this interaction, which has been implicated in obesity and NAFLD (17).

Some papers believe that high carbohydrate intake is responsible in the development of this disease (18-20); while other researches provide documents against high fat diets (21-24). It has been well established that excessive serum levels of free fatty acid (FFA), glucose and insulin lead to higher fat storage in hepatocytes, due to inducing insulin resistance (25). Some recent papers emphasis on the kind of fat intake and discuss on different kinds of fat (26, 27). As there are some challenges regarding the role of macronutrients (18-24, 26-31), we discussed the evidence regarding the macronutrients intake with the development and treatment of NAFLD in the present review article.

## 2. Evidence Acquisition

The relevant English and non-English published papers were searched using PubMed, ISI Web of Science, SCOPUS, Science Direct and EMBASE from January 2000 to January 2013. We used the following keywords in our search: non-alcoholic fatty liver, non-alcoholic steatosis fatty liver, macronutrients, carbohydrate, fat and protein. We found 1575803 English papers published between 2000- and 2013; however, most were excluded because some of them had molecular and biochemical view, and others investigated animal model without any practical and clinical approach or general point regarding the lifestyle and diet without any specific focus on macronutrients. Finally, we included 40 papers in this study. We also summarized the most important studies reviewed in the current paper in Table 1. 

**Table 1.** A Summary of the Most Important Papers Reviewed in the Current Paper

Reference/ Year	Subjects/ Country	The Proportion of Macronutrients	The Duration of Intervention	Liver Fat Assessment	Findings
**Utzschneider KM (32)/ 2012**	Parallel and randomized clinical trial/ 35 subjects / USA	isoenergetic low-fat/low-saturated fat/LGI^a^ (LSAT ^a^: 23% fat/7% saturated fat/GI ^a^< 55) or a high-fat/high-saturated fat/high-GI (HSAT: 43% fat/24% saturated fat/GI > 70)	4 weeks	Magnetic resonance spectroscopy	LSAT decreased significantly A liver fat (median 2.2 (IQR ^a^ 3.1) to 1.7 (IQR 1.8) %) but HSAT ^a^ did not (median 1.2 (IQR 4.1) to 1.6 (IQR 3.9) %).
**Zelber-Sagi S (33)/ 2007**	Cross-sectional study/ 349 volunteers/ Israel				NAFLD ^a^ group consumed less fish (the rich source of omega-3) and more amount of soft drinks and meat rather than healthy population.
**Machado RM (34)/ 2010**	Parallel clinical trial/ 41 male mice/ Brazil	Diets containing 40% energy as either trans fatty acid (TRANS), PUFA, or SFA.	16 weeks	Histological markers of NAFLD	compared with PUFA- and SFA-fed mice, TRANS-fed mice had NASH-like lesions
**Shertzer HG (35)/ 2011**	Parallel clinical trial/ 4 Female mice/ USA	A HF ^a^ diet, with or without 100 g Whey Protein Isolate/L drinking water	11 weeks	Histological markers	Livers from WPI mice had significantly fewer hepatic lipid droplet numbers and less deposition of nonpolar lipids.
**Yang HY (36)/ 2011**	Parallel clinical trial/ 40 male Sprague-Dawley rats/ Taiwan	A NASH-inducing diet, a standard diet, a NASH-inducing diet plus soy protein, and a standard diet plus soy protein.	10 weeks	Histological markers	Soy protein intake decreased the hepatic lipid depots of triacylglycerols
**van der Meer R W (23)/2008**	Before-after/ 15 healthy males/ Netherlands	HFHE ^a^ diet: A regular diet, supplemented with 800 mL cream (280g fat) every day	3 days	H-magnetic resonance spectroscopy	The HFHE diet increased hepatic TGs compared tobaseline (from 2.01 ± 1.79 to 4.26 ± 2.78%)
**Westerbacka J (24)/ 2005**	Randomized Cross-over/ 10 overweight apparently healthy premenopausal women/ Finland	Isocaloric diet	2 weeks	Magnetic resonance proton spectroscopy	Liver fat decreased by 20 ± 9% during the low-fat diet and increased by 35 ± 21% during the high-fat diet
		-High fat diet: Fat: 56 ± 1%, CHO:31 ± 1%, Protein: 13± 1%, SFA ^a^: 28 ± 1%, MUFA ^a^: 16 ± 1%, PUFA ^a^: 5 ± 1%			
		-low fat diet: fat: 16 ± 1%, CHO: 61 ± 3%, Protein: 19 ± 1%, SFA: 5 ± 1%, MUFA: 5 ± 1%, PUFA: 3 ± 1%			

^a^ Abbreviations: GI, glycemic index; HF, high fat; HFHE, high fat high energy; HSAT, high saturated fatty acid; IQR, interquartile range; LGI, low-glycaemic index; LSAT, low saturated fatty acid; MUFA, mono unsaturated fatty acids; NAFLD, non-alcoholic fatty liver disease; PUFA, poly unsaturated fatty acids; SFA, saturated fatty acids.

## 3. Results

### 3.1. Carbohydrate intake and NAFLD

Previously, a low fat diet was considered as an optimal suggestion for NAFLD. However, recent studies revealed that low carbohydrate diets have more beneficial effects in these patients. High-carbohydrate diets contribute to the association between hepatic steatosis and insulin resistance through activation of the transcription factor ChREBP (Carbohydrate response element binding protein). Postprandial hyperglycaemia raises the hepatic concentrations of phosphorylated intermediates causing activation of ChREBP and induction of its target genes. Both enzymes of glycolysis and lipogenesis pathways and glucose 6-phosphatase (G6PC) are involved, which may result in insulin resistance. High-carbohydrate diets induce hepatic insulin resistance to protect the liver from substrate overload (18). According to the recent reports, patients with NAFLD should intake a low carbohydrate and low saturated fat diet; avoid fructose-enriched soft drinks and consume more fruits and vegetables (19).

A thirty-six months cohort study compared soft drink consumption between healthy and NAFLD patients without classic risk factors such as obesity, diabetes and insulin resistance (37). Different soft drinks including Coca-Cola sweetened with 55% free fructose, diet Coke sweetened with aspartame and flavored fruit juice colored with caramel were assessed in this study. Findings showed a correlation between the severity of fatty liver and the amount of soft drink consumption (dose-response), and authors introduced soft drink as an independent risk factor for fatty liver. Although it might be inferred that higher consumption of soft drinks associated with higher intake of sugar and calorie (38, 39), in the Assy’s study, the amount of energy and macronutrients were similar between healthy and NAFLDs. Therefore, they suggested more risk factors for NAFLD by soft drinks such as fructose, aspartame and caramel. A probable mechanism for increasing the risk of NAFLD by aspartame might be due to mitochondrial dysfunction and ATP depletion in liver because of aspartame metabolism (40). However, to the best of our knowledge, no study has assessed the association of aspartame and other artificial sweeteners with NAFLD in humans. Fructose not only contributes to hepatic steatosis; but also triggers inflammatory signals as well and plays a role in second “hit” of fat injury (16, 31, 41). Several factors including the induction of metabolic syndrome, copper deficiency, formation of advanced glycation end products and a direct dysmetabolic effect on liver enzymes may potentially contribute to fructose-induced NAFLD (31). Furthermore, a recent before-after study showed that a 50% reduction of fructose intake along with a moderate weight reduction reduce intrahepatic fat content, after 6 months (42).

However, most of experimental studies tend to include high intake of fructose (60-70% of total energy intake) which is not reflective of average human intake. Combining in vivo, in vitro and genetic researches would provide substantial mechanistic evidence into the role of fructose in NAFLD development and its complications (31). Some mechanisms underlying the pathogenesis of NAFLD by fructose rely on its ability to alter intestinal microbiota (43), increase portal vein endotoxin and hepatic TNF-α and TG (43), and copper metabolism alteration (44).

Carbohydrate quantity and quality has a role in NFALD. A recent paper showed the beneficial effect of a low-fat/low-saturated fat/low-glycemic index diet to reduce liver fat in older subjects (32). A quasi-randomized controlled trial compared the effects of 3 different diets in patients with obesity and type 2 diabetes (45). Prescribed diets were: 1) the 2003 recommended American Diabetes Association diet (ADA) containing 50-55% carbohydrate, 30% fat, and 20% protein, 2) a low glycemic diet which was similar to ADA diet in macronutrients content, but varied in the kind of carbohydrate, and 3) a modified Mediterranean diet which contained 35% low glycemic index carbohydrate, 45% fat high in MUFA content, and 20% protein. Individuals in modified Mediterranean diet had the lowest alanine aminotransferase levels either after 6 or 12 months. These findings remained significant even after adjustment for insulin resistance, triacylglycerol and anthropometric measures. Although, exercise and dietary content of sodium, potassium, magnesium, calcium and energy were controlled in the three diets; it was impossible to determine which change (low content of carbohydrate or high content of MUFA) leads to the observed effects. However, a recent longitudinal study showed that the changes in the quality of dietary carbohydrate (glycemic index, sugar, starch and fiber intake) were not related to the changes in liver enzymes (ALT and γ-glutamyltransferase (GGT)) after 5 years (46).

### 3.2. Fat Intake and NAFLD

Several studies in animal and human models have shown that a high-fat diet induces hepatic steatosis (21, 22, 43). A five-day trial showed that an isoenergetic high-fat, low-carbohydrate diet causes a 3.7-5.3-fold increment in the liver de novo lipogenesis in hyperinsulinemic obese patients compared to normoinsulinemic lean and obese subjects (47). However, few studies are available among humans.

According to the reports, different kinds of fat can act differently in non-alcoholic fatty liver. Saturated fat, monounsaturated fat, polyunsaturated fat, omega-3 fatty acids, and all-trans fat have different roles in NAFLD. Increased saturated fatty acid intake (SFA) is usually associated with high insulin resistance which can provoke NAFLD progression. Evidence shows that SFA accumulation in the liver can have detrimental effects on the liver function. An epidemiological study revealed that the ratio of polyunsaturated/saturated fatty acid intake in both the NASH and fatty liver patients was lower than the ratio in randomly selected controls (27). However, due to the results of few studies, limited amount of SFA may prevent insulin sensitivity. Therefore, high amount of saturated fatty acid intake (more than 7% of calorie intake) should be avoided for preventing NAFLD. While, lower limitation may have no further beneficial effects. The results regarding cholesterol intake are controversial. Some reports confirmed no difference between cholesterol intake among NAFLD patients and control group (29, 33).

Essential fatty acid deficiency could be associated with liver steatosis in the rat model studies. Polyunsaturated fatty acids with omega-6 and omega-3 fatty acids have crucial role on cardiovascular risks. Omega-3 fatty acids intake is also related to reduce insulin sensitivity. It seems that n-3 fatty acids found in fish oils and walnuts may improve blood lipid profiles and reduce inflammation, steatosis, and liver damage in patients with NAFLD. A 8-week randomized cross-over clinical trial found that dietary supplementation with 4 g/d omega-3 fatty acids decreased liver fat content without any significant change in serum ALT concentration in women with polycystic ovary syndrome (PCOS) (48). In a current systematic review, Parker et al.(49) reported a beneficial effect of omega-3 supplementation on liver fat and AST (49), but the optimal dose was unknown. In another study, the prolong omega-3 supplementation effect on hepatic steatosis was assessed (50). Findings showed that one g/d omega-3 consumption for 12 months improves ultrasonographic and haemodynamic features of liver steatosis. Rat models studies showed that diets enriched with n-3 PUFA increase insulin sensitivity (26), reduce intrahepatictriglyceride content, and ameliorate steatohepatitis (51). Polyunsaturated fatty acids are also thought to exert anti-inflammatory effects (16). The effect of n-3 fatty acids on lipid metabolism was mediated via genomic pathways and they regulate the transcription of involving genes in the lipid metabolism such as peroxisome proliferator-activated receptor-γ (PPARG), sterol regulatory element binding protein-1 (SREBP-1), and the carbohydrate regulatory element binding protein (CREBP) (52). Additionally, decreased postprandial lipemia and increased activity of lipoprotein lipase by omega-3 fatty acids reduce the plasma pool of triacylglycerol and thereby the liver uptake of triglycerides from the circulation (53). However, data on omega-6 fatty acids are very limited and mainly restricted to animal models.

Besides PUFAs, dietary MUFAs also have beneficial effects on NAFLD. MUFAs could decrease oxidized LDL, LDL cholesterol, total cholesterol (TC), and triacylglycerol concentrations; without decreasing HDL, which is usually provided by low-fat diets. It is shown that the replacement of carbohydrate and saturated fat with MUFAs results in glucose and blood pressure reduction and serum HDL increase. It is reported that a MUFA-rich diet (40% of energy as fat) is more acceptable than a high-carbohydrate diet (28% of energy as fat) for patients with diabetes (54). A recent study by Bozzetto (55) compared the effects of four different isocaloric intervention programs on liver fat in patients with type 2 diabetes. Subjects were randomly assigned to consume one of the following diets: 1) high carbohydrate/high fiber/low GI (CHO/fiber group); 2) high MUFA diet (MUFA group); 3) high carbohydrate/high fiber/low GI plus exercise (CHO/PA group); and 4) high MUFA diet plus exercise (MUFA/PA group). The proportions of SFA, PUFA and protein were similar in all four diets. After an 8-week period, MUFA rich diet compared with high CHO/high fiber/low GI diet reduced hepatic fat independent of aerobic exercise. Therefore, MUFAs replacement instead of SFAs and carbohydrate may be beneficial for patients with NAFLD. The probable mechanism for MUFA beneficial effect on liver fat content might be related to its expression regulation potency of involved genes in peripheral insulin sensitivity (56), anti-inflammatory (57) and inhibitory effects on nuclear factor- κB (NF-κB) (58). In a study, MUFA decreased the expression of hepatic lipogenesis and gluconeogenesis genes and SREBP in fatty rats (59).

Nowadays we have a problem with all-trans fats produced by hydrogenation. There are some natural all-trans fats in dairy products and different kinds of meat; however, the detrimental effects of all-trans fats are mostly related to those all-trans fats that are the results of hydrogenation of liquid vegetable oils. Partially hydrogenated vegetable oils are the most prevalent source of all-trans fats due to the hydrogenation process. Trans fats had the most harmful effects on the lipid profiles and insulin resistance according to the recent publications (60).

According to a recent paper, compared with PUFA- and SFA-fed mice, TRANS-fed mice had less adiposity, impaired glucose tolerance characterized by greater HOMA (IR) index, and NASH-like lesions due to greater hepatic lipogenesis (34). The mechanism of developing of NASH in all-trans-fed mice attributed to higher gene expression of SREBP-1c and PPAR-γ rather than PUFA- and SFA-fed mice. Furthermore, TG releasing from liver was decreased in all-trans-fed mice compared with PUFA-fed ones, due to inadequate transferring of TG to nascent ApoB particles. However, in this study, no significant difference was observed in the liver capacity of β-oxidation among the three intervention groups (34).

### 3.3. Fat or Carbohydrate; Which One is Important in the Diet of People in the Middle East?

Carbohydrate involves the major dietary components in the Middle East (61). White rice, refined white bread and cereals are the most important staple foods in the Asian countries (61). In Iran, both the diversity and amount of whole grain products are limited (62); while, the amount of fat intake may not be a matter of concern (63). However, more problems on fat intake are related to the type of fat intake, e.g. saturated fat intake or all-trans-fat consumption due to partially hydrogenated fat intake (63). Regarding carbohydrate intake in the Asian countries, problem is related to both kind and amount of carbohydrate consumption (64). Nutrition transition in these countries provides higher intake of simple sugars and refined carbohydrate. On the other hand, western dietary pattern is characterized by high refined grains, red meat, butter, processed meat, high-fat dairy products, sweets and desserts, hydrogenated fats, and soft drinks and low vegetables, and low fat dairy products consumption is a major dietary pattern in our country. Furthermore, western dietary pattern is positively associated with abdominal obesity, inflammation, dyslipidemia (65-67) and consequently might be a risk factor for NAFLD. However, changing dietary habits to reduce carbohydrate and fat intake or replacing them with healthy foods would not be easily achieved. Although Iranian diets contain high saturated fatty acids, only around 25% of dietary energy intake was provided by fat, while around 64% of energy intake was obtained from carbohydrate, specially simple and refined carbohydrate (68). Hence, it seems that in our country, the amount and type of carbohydrate is the main concern for diet therapy in patients with NAFLD. Furthermore, we should consider the detrimental effects of high GI or high GL diets on metabolic parameters (69). White rice which has a high GI and GL is consumed in high amount in the Asian countries (32); which may be associated with unfavorable metabolic profiles in these countries (69). Following nutrition transition in the Asian countries, both quality and quantity of dietary carbohydrates, and need great attention. Although, the Asian countries may have lower problems regarding the amount of fat intake, it also needs to be considered after convenient and fast foods enter their society. However, according to the evidence, quantity and quality of carbohydrates may be the first concerns in these countries.

### 3.4. Protein Intake and NAFLD

The effects of protein quality and quantity on NAFLD have been poorly assessed and these studies are mainly limited to animal models. An increment in dietary protein content has been shown to reduce the risk of hepatic fat accumulation during a high fat diet both in human and rodents (35, 70, 71). Likewise, protein malnutrition leads to steatosis (72, 73). Some animal models provide cellular mechanisms underlying the favorable effects of low CHO: protein ratio on fatty liver in rats (74). They showed that a high protein diet reduces gene expression of stearoyl-CoA desaturase-1 (SCD-1) which is positively correlated with plasma TG concentrations and conversely upregulates fibroblast growth factor-21 (FGF21) gene expression, which modulates the expression of lipolysis and lipid utilization genes in liver (74). On the other hand, FGF21 and SCD-1 regulation, by a low CHO: protein ratio diet, stimulate hepatic lipid oxidation and utilization. Another study in mice found that those consuming high protein diets (either low or high fat) had significantly lower hepatic fat accumulation than did those consuming either low fat-normal protein diet or high fat-normal protein diet after 1 or 12 weeks (71). A well-designed trial showed that healthy subjects lost 22% intra hepatocellular lipids (IHCLs) deposition (70) after a high protein-high fat diet (% protein of total calorie = 23%) compared with high fat and control diets (% protein of total calorie = 11%). However, this study was performed during 4 days and the findings may be different after a long term. The quantity and type of protein is another concern which has been poorly assessed. Available evidence suggests that whey protein and soy protein could prevent or ameliorate fatty liver (35, 36), but these studies are exclusively limited to animals and to the best of our knowledge, there is no study in human. The lower feeding efficiency by whey protein consumption may be one of the reasons of its protective effect on liver; since whey protein consumption was associated with higher basal metabolic rates and mitochondrial oxygen consumption and lower dietary lipid utilization (35). Reduced oxidative stress and systemic inflammation might be another possible mechanism achieved by whey and derived-whey proteins (75). Moreover, the high content of branched chain amino acids (BCAAs) in whey products modifies the gene expression leading to more β-oxidation of fatty acids and protects pancreatic β-cells against AMP activated kinase-mediated apoptosis (76). However, the exact mechanisms underlying the less efficiency of amino acids for transferring to fat in liver remain to be elucidated. It seems that high protein intake is associated with higher catabolic rate of amino acids, which takes place in liver by consuming a large amount of energy. Higher energy consumption for amino acid catabolism increases energy expenditure and lipid oxidation in liver and prevents fat accumulation in hepatic cells (77). Furthermore, the augmented level of bile acids by protein consumption inhibits lipogenesis (78); while, increased day-long releasing of glucagon stimulates ketogenesis in liver (79). Altogether, there are very few studies assessing the effects of protein consumption and related mechanisms on hepatic fat storage. Available studies were performed in a short periodor in animal models. Conducting more studies to assess the effects of quality and quantity of protein on fatty liver disease is necessary.

## 4. Conclusions

Dietary treatment of NAFLD and NASH should be highly individualized based on nutritional status, dietary habits, and personal goals and preferences. The replacement of macronutrients with each other should be resulted in acceptable diets by patients. It is important to choose an appropriate replacement for restricted macronutrients since they could affect the compliance of recommended diet. Due to the close associations between obesity, metabolic syndrome and various metabolic abnormalities, especially insulin resistance and NAFLD, it is probable that patients with NAFLD face metabolic abnormalities. Hence, nutrition therapy for liver diseases should be consistent with dietary recommendation which improves these abnormalities. Although, weight reduction and lifestyle changes such as exercise and dietary intakes are considered as the main treatment goals for NAFLD, there is very few evidence on macronutrients and NAFLD, especially in human models. Higher consumption of simple carbohydrate, sweetened drinks and saturated fatty acids may exacerbate NAFLD and cause more fat accumulation in the liver; whilst higher intake of fiber and low GI carbohydrate, MUFA and n-3 fatty acids, soy and whey protein tend to be favorable to ameliorate NAFLD. Moreover, it seems that low carbohydrate, low fat and high protein content of diets are helpful for patients with NAFLD. Fraser study (45) showed that replacement of carbohydrate (a high fiber and low GI diet) with MUFA has greater beneficial effects on NAFLD. High consumption of refined carbohydrate might be the main concern regarding fatty liver in our country, which should be corrected. However, due to nutrition transition and a growing trend in fast foods consumption and consequently increased all-trans and saturated fatty acids intake, it is necessary to consider fat consumption patterns of Iranians, and therefore, the efficacy of such diets (low carbohydrate-high MUFA) should be assessed in Iranian patients. 

### 4.1. New Aspects of the Current Review

To the best of our knowledge, the present review was the first article; which reviewed and compared the effects of various macronutrients intake in NAFLD. Previous review articles (20, 60, 80) focused on the pathogenesis of NAFLD (80), or discussed the low carbohydrate and ketogenic diets (21) or the dietary recommendations for MetS beside the NAFLD (57). Although, we declared that dietary recommendations for NAFLD are the same for MetS, Zivkovic (60) in his study did not discuss the outcomes of replacing carbohydrate and with each other. Furthermore, they poorly discussed dietary protein intake and did not provide any comparison between different countries with different dietary patterns and macronutrient profile.

### 4.2. Limitations and Future Directions

Due to the lack of evidences for proper dietary approach, there is no consensus on diet therapy in patients with NAFLD. For example, the favorable percentage of different macronutrients for patients with NAFLD has not been suggested; furthermore, the optimal dose of omega-3 fatty acids, proper candidate for n-3 PUFA (DHA or EPA), adequate time for supplementation and the safe proportion of different types of fatty acids have not been clarified. Another limitation regarding the fatty acids is their long-term effect on liver fat storage. On the other hand, few studies have assessed the effect of omega-3 fatty acids on prevention of fibrosis and cirrhosis. Moreover, the permissible amount of refined carbohydrate or soft drinks and fructose and the safety of artificial sweeteners for liver are questionable. Most of available studies are limited to assess the effects of replacing carbohydrate and fat with each other. Nevertheless, both high and low protein intakes are associated with liver damage. Hence, future studies should be conducted to assess the effects of dietary protein quality and quantity on NAFLDs, when consumed as the replacement of dietary fat or carbohydrate. Additionally, many studies conducted to assess the effects of n-3 PUFA on NAFLD, but only few studies assessed the effects of dietary n-6 PUFA and cholesterol intake on liver function. Furthermore, it is beneficial to assess health outcomes of various vegetable oils on liver because of their different antioxidants content and consequently their different metabolic outcomes. Another limitation in this field was related to the period of conducted studies. Most of researchers reported their clinical trials results after a short period. Well-designed randomized clinical trials and long-term longitudinal studies are needed for quantify the optimal proportion of various macronutrients in patients with NAFLD.
